# Herbal Extracts Combination (WNK) Prevents Decline in Spatial Learning and Memory in APP/PS1 Mice through Improvement of Hippocampal A****β**** Plaque Formation, Histopathology, and Ultrastructure

**DOI:** 10.1155/2012/478190

**Published:** 2012-07-02

**Authors:** Wei-hong Cong, Bin Yang, Li Xu, Xiao-xia Dong, Li-song Sheng, Jin-cai Hou, Jian-xun Liu

**Affiliations:** Research Center, Xiyuan Hospital, China Academy of Chinese Medical Sciences, Beijing 100091, China

## Abstract

To investigate the cognitive enhancement effect of WNK, an extracts combination of *P. ginseng*,  *G. biloba*, and *C. sativus* L. and possible mechanisms, 5-month-old APP/PS1 transgenic mice were used in this study. After 3 months of administration, all mice received Morris water maze (MWM) training and a probe test. Mouse brain sections were detected by immunohistochemistry, HE staining, and transmission electron microscopy. MWM results showed significant difference between transgenic mice and nontransgenic littermates (*P* < 0.05, *P* < 0.01). WNK-treated mice exhibited enhanced maze performance over the training progression, especially better spatial memory retention in probe test compared to transgenic mice (*P* < 0.05, *P* < 0.01) and better spatial learning and memory at the fourth day of MWM test compared to EGB761- (*G. biloba* extract-) treated ones (*P* < 0.05). Hippocampal A**β** plaque burden significantly differed between APP/PS1 and littermate mice (*P* < 0.001), while decreased A**β** plaque appeared in WNK- or EGB761-treated transgenic brains (*P* < 0.05). Neurodegenerative changes were evident from light microscopic and ultrastructural observations in transgenic brains, which were improved by WNK or EGB761 treatment. These data indicate WNK can reduce the decline in spatial cognition, which might be due to its effects on reducing A**β** plaque formation and ameliorating histopathology and ultrastructure in hippocampus of APP/PS1 mouse brain.

## 1. Introduction

Neuropathological examination of the Alzheimer's disease (AD) brain shows extensive neuronal loss, accumulation of fibrillar proteins as extracellular *β*-amyloid (A*β*) plaques, and as neurofibrillary tangles (NFTs) inside neurons [1]. Autosomal dominant AD has been proved to be associated with mutations of APP, PS1, and PS2 genes [2]. These discoveries permitted researchers to work with some transgenic models of AD, such as APP and APP/PS1 transgenic mice. In the present study, APP/PS1 double transgenic mice overexpressing chimeric mouse/human APP with the Swedish double mutation (K595N and M596L) and human PS1-ΔE9 (deletion of presenilin 1 exon 9) mutation were used [3, 4]. This line is originally maintained in a hybrid C3HeJ C57BL6/J F1 background and manifests a rapid accumulation of amyloid plaques in the cortex and hippocampus beginning at about 3 months of age [4–7]. Additionally, these mice exhibit other pathological featured alterations associated with AD [8, 9].

Unfortunately, there have been few safe and effective methods for the treatment or prevention of AD at present. Given the situation, the use of natural medicinal plants and traditional Chinese medicines as remedies cast new light on some chronic or complicated disorders, such as AD, which involves many unknown mechanisms and various systems in the pathogenesis. WNK is an extracts combination of three traditional Chinese herbal medicines: *P. ginseng*, *G. biloba*, and **C. sativus** L., which have been used as traditional Chinese treatments for several millennia for the treatment of a variety of conditions, including age-related memory decline. In recent years, they have also become increasingly popular throughout Western society, both as “over-the-counter” herbal supplements and as prescription drugs [10, 11]. Over recent years, especially, certain active constituents isolated from them have been proved to be essential to cognitive efficacy in several paradigms. For example, ginsenosides, ginseng's main active ingredient, can enhance psychomotor and cognitive performance and can benefit AD by improving brain cholinergic function, reducing the level of A*β* and repairing damaged neuronal networks [12, 13]. Evidence suggests that chronic administration of the standardized *G. biloba* extracts (EGB761), containing 24% of Ginkgo glycosides and 6% of ginkgolides-bilobalide, can ameliorate the cognitive decline that occurs during ageing and may also play a beneficial role in the attenuation of the cognitive deficits associated with a number of pathological conditions, including intermittent claudication, vascular dementia, AD, and cerebral insufficiency—a generalised condition with a presumed cerebrovascular aetiology [14–19]. *C. sativus* L., commonly known as saffron, is used in traditional Chinese medicine as a nerve sedative, antispasmodic, anticatarrhal, stimulant, and so forth. Modern pharmacological studies have demonstrated that saffron or its active constituents has memory-improved, A*β* aggregation-inhibited, radical scavenging, hypolipaemic, and antitumour effects [20–27]. Improvements in memory performance have also been demonstrated preclinically in both young and old rats, as well as in AD rat models, and clinically in healthy middle-aged and neurasthenic human cohorts, following chronic administration of ginseng, ginkgo, and their combination [28–30].

However, it seems still vague of the current overall evidence that saffron has a predictable and clinically significant benefit for people with dementia or cognitive impairment. Same situation occurred to ginkgo, although its extract is among the most widely used complementary therapies today [31, 32]. In the present study, we investigated the effect and the possible mechanisms of action of WNK in an APP/PS1 transgenic mouse model of AD. The effects of WNK and EGB761 on maze performance were compared simultaneously.

## 2. Materials and Methods

### 2.1. Animal

Animals of both sexes were used in this study. The transgenic APP/PS1 mice, which expressed both the Swedish double mutations of *APP *(K670N/M671L) and mutant *PS1* (PS1M146L), were provided by the Institute of Laboratory Animal Science, Chinese Academy of Medical Sciences, and Peking Union Medical College, Beijing, China. APP695SWE/co + PS1/ΔE9 transgenic (*n* = 29) and littermate wild-type mice (*n* = 10), controlled for age (5 months, range 4–6), were separated into 4 groups: APP/PS1+tap water (transgenic group), *n* = 9; APP/PS1 + WNK 44 mg/kg/day (WNK group), *n* = 10; EGB761 30 mg/kg/day (EGB761 group), *n* = 10; nontransgenic littermates + tap water (nontransgenic group), *n* = 10. There was no intergroup difference in body weight (*P* > 0.05, data not shown).

Mice were fed in separate home cages with free access to water in the special animal room under a 12 : 12 h light/dark cycle and thermoregulated environment. The mice were acclimatized to laboratory conditions before the behavioural test. All the procedures took place between 8 AM up to 4 PM, and each mouse was used only one time in the water maze. All experiments were conducted according to the guidelines for care and use of animals approved by Xiyuan Hospital, China Academy of Chinese Medical Sciences, China.

### 2.2. Chemicals and Reagents

EGB761 (batch no.: c02191), the *G. biloba* leaves extract tablets (EGb761), was product of Beaufour Ipsen Industrie, Dreux, France. Rabbit anti-human A*β* antibody was purchased from Biosynthesis Biotechnology, Beijing, China. Goat anti-rabbit IgG antibody was from Abcam, Cambridge, MA, USA. Ethanol, formaldehyde, and other reagents used in this study were all HPLC-grade obtained from Fisher Scientific, New Jerseys, USA.

### 2.3. Formulation of WNK and Concentration Control of Active Components

WNK (Wei Nao Kang) is a proprietary formula (China: ZL02131435.7; Russia: 2008144409/21(057916); Australia: 2006342350, etc.) consisting of extracts of three traditional Chinese herbs,* P. ginseng*, *G. biloba*, and saffron, that is, total ginsenosides, total gingkgo flavonoids, and total saffron glycosides. WNK's dose used in this study was obtained according to the results of two independent orthogonal designs with animal models of D-galactose-injected mice and focal cerebral ischemia-reperfusion rats (data not shown). In this study, the combination of total ginsenosides (batch no.: 100909), total ginkgo flavonoids (batch no.: 100914), total saffron glycosides (batch no.: 100910), was provided by Shineway Pharmaceutical Group, Hebei, China. HPLC and UV were used to analyze and control the active components of WNK (Table 1).

### 2.4. Drug Administration

Mice of WNK or EGB761 groups were administered with WNK (44 mg/kg/day) or EGB761 (30 mg/kg/day) intragastrically in a volume of 10 mL/kg once a day for 3 months. Mice of nontransgenic and transgenic groups were given tap water with the same volume.

### 2.5. Behavioural Assessment

Since the Morris water maze (MWM) test evaluating spatial learning has been shown to be sensitive to APP/PS1 transgenic models, it was used to assess hippocampal-dependent spatial learning and memory in this study [33–35].

The water maze was a white circular pool (80 cm in diameter, 50 cm deep) divided into four equal imaginary quadrants for data analysis. The water temperature was maintained between 21 and 23°C. One centimeter beneath the surface of the water and hidden from mouse view was a white circular platform 10 cm in diameter. The swimming patterns of the mice were recorded with a video camera mounted above the center of the pool and analyzed with a video-tracking system. The water maze was located in a room with several visual stimuli hanging on the walls to provide spatial cues. The water in the pool contained dark ink so that the mice could not see the platform. The time mice spent on finding the platform was recorded as the escape latency. Every mouse has 4 times of swimming each day, released at the first, second, third, and fourth quadrant, respectively. Mice not finding the platform within 180 s were guided to it by the experimenter. This part of test was repeated for 4 days, and the location of the platform was not changed. At the fifth day, the platform was removed and the mice were released at the first quadrant. The time spent swimming in the target quadrant (where the platform was located during hidden platform training) was considered as a measure of platform location retention.

### 2.6. Histopathology

One day after behavioral analysis, mice in different groups were anesthetized with 5% chloral hydrate and perfused through the left ventricle with 0.9% saline. The brain was removed from the skull and then postfixed for 24 h with 4% paraformaldehyde. After being progressive dehydrated, the brain was embedded in paraffins. Four *μ*m thick consecutive coronal paraffin sections were collected throughout the cerebral cortex and hippocampus, according to the Mouse Brain in Stereotaxic Coordinates. Each group consisted of three mice. Hematoxylin and eosin (HE) sections were obtained every 20 sections. Photomicroscopy was done with an Olympus BX41 light microscope and Anymicro DSS Image Analysis software. Exposure settings were identical among sections per magnification using ×400 objectives. Once linear best-fit setting was identified, this was used for all sections per magnification and stain type. Minimal image adjustments were applied to match the actual image when viewed directly through the eyepiece.

### 2.7. Immunohistochemistry

Mice in different groups were anesthetized with 5% chloral hydrate and perfused with saline and then 4% paraformaldehyde through the left ventricle. Serial 4 *μ*m thick paraffin sections were cut throughout hippocampus and cortex according to The Mouse Brain Atlas (Paxinos and Franklin, 2001) and mounted on APES-coated slides. Sections were deparaffinized in xylol, followed by heat-mediated antigen retrieval in 10 mM citrate buffer (pH = 6.0) and incubation with 3% H_2_O_2_ for 40 min at 37°C. Sections were then incubated with 10% normal goat serum for 20 min at 37°C to block the nonspecific binding followed by incubation overnight at 4°C with antibody against A*β* (1 : 300). Sections were washed in 0.1 M PBS, incubated sequentially with fluorescent-labelled secondary antibody, goat anti-rabbit IgG (1 : 200) for 45 min at 37°C. Fluorescent signals were detected using an Olympus 1 × 81 microscope with a Rolera-MGi Plus back-illuminated EMCCD camera (Qimaging, Surrey, Canada) using identical exposure times. Controls received identical treatment except that primary antibody was omitted and showed no specific staining. Intensity was detected by Image-Pro Plus (In Vivo Version 6.0, MediaCybernetics Inc.) using ROIs.

### 2.8. Ultrastructure

The brains were removed immediately from the skull, pieces of selected brain regions (hippocampus CA1 area) were separated and immediately placed in 2.5% glutaraldehyde, chopped to get pieces of about 1 mm^2^, and kept in 2.5% glutaraldehyde for more than 12 h at room temperature. Samples were postfixed in 1% osmium tetroxide for 1 h, dehydrated in graded increasing ethanol, and embedded in epoxy resin. Polymerization was performed at 80°C for 24 h. Blocks were cut on a Reichert ultramicrotome into ultrathin sections (60–70 nm), which were poststained with uranyl acetate and lead citrate, and viewed under a Hitachi 7100 electron microscopy.

### 2.9. Statistical Analysis

All data were expressed as mean ± SD, and statistical significance was evaluated by a one-way ANOVA or *t*-test. The data of escape latency in behavioral testing was analysed using repeated measures of ANOVA analyze. Values with *P* < 0.05 were considered significant for all the analysis.

## 3. Results

### 3.1. Learning and Memory Performance Enhancement

**Figure 1:**
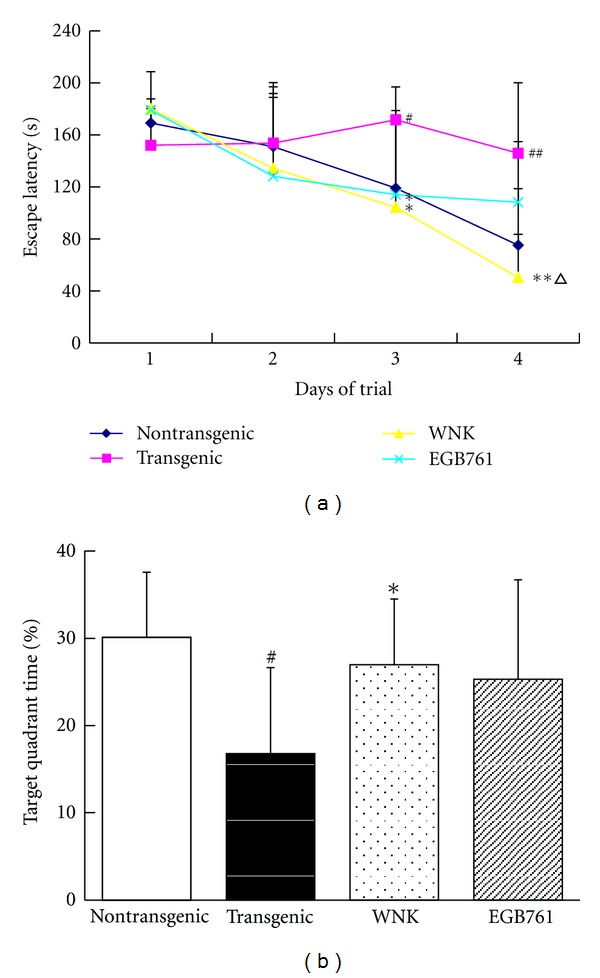
Effect of WNK on spatial learning and memory in APP/PS1 mice. (a) Escape latency to reach hidden platform in Morris water maze; (b) percentage of time spent in target quadrant of Morris water maze. Note: ^#^ *P* < 0.05, ^##^ *P* < 0.01, compared with nontransgenic group; *P < 0.05, **P < 0.01, compared with transgenic group; ^∆^ *P* < 0.05, compared with EGB761 group.

The mean escape latency was recorded from four quadrants in one day. During the course of the experiment, the mean latency of nontransgenic, WNK- and EGB761-treated mice in finding the hidden platform became shorter from the first day to the fourth day. The results showed significant difference between transgenic mice and their nontransgenic littermates (*P* < 0.05, *P* < 0.01), and the differences among WNK- or EGB761-treated mice and transgenic mice were also significant (*P* < 0.05, *P* < 0.01), especially at the final day of the invisible platform task. Besides, the results indicated that the learning ability was different between WNK and EGB761-treated mice, since the latency was found decreased to a higher degree in WNK group at the fourth day (*P* < 0.05; Figure 1(a)).

The time that mice swam in the first quadrant, the previous platform location, at the fifth day indicates the memory ability. In this study, the time that the transgenic mice swam in the target quadrant was significantly shorter than that of the nontransgenic mice (*P* < 0.05), while it was significantly longer, compared the WNK-treated mice with the transgenic ones (*P* < 0.05). No significant difference was shown between transgenic and EGB761-treated mice (*P* > 0.05; Figure 1(b)).

### 3.2. Histopathological Observation

**Figure 2:**
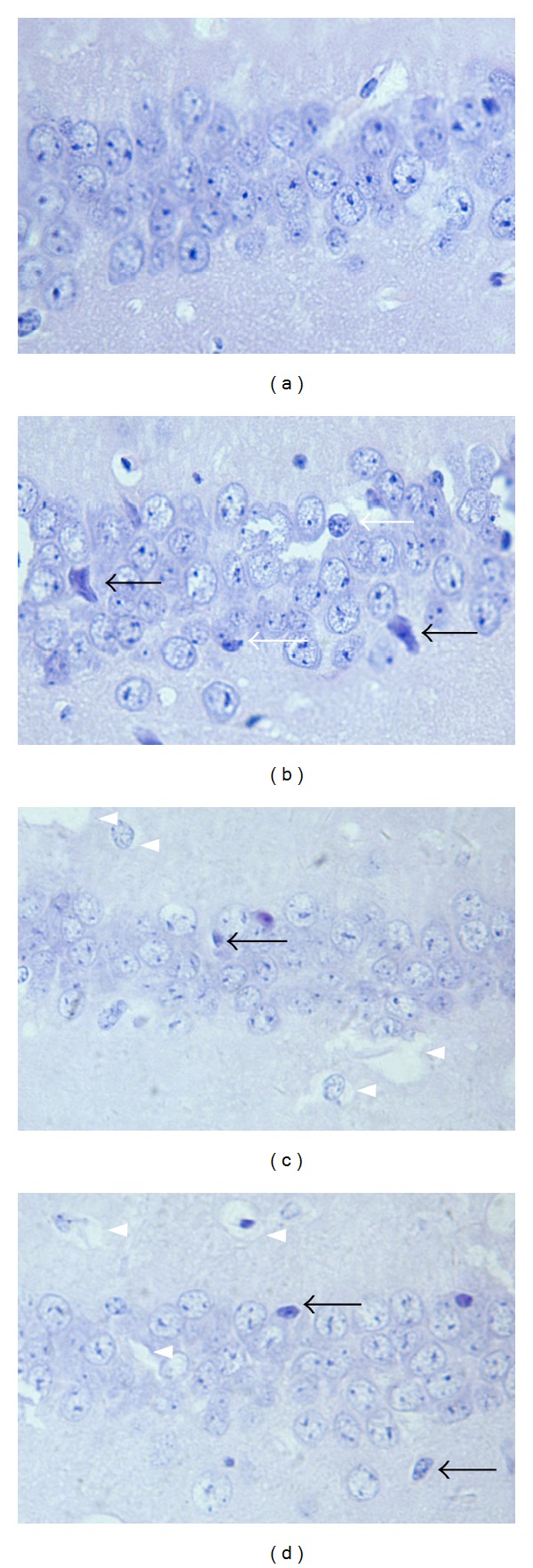
Light micrographs of HE-stained CA1 hippocampal neurons (×400). (a) Nontransgenic group; (b) transgenic group; (c) WNK group; (d): EGB761 group. Neurons in nontransgenic mouce brain exhibited well-defined shapes and nucleus (Figure 2(a)). Damaged neurons in transgenic one are darkly stained and exhibited shrunken and triangulated neuronal body (black arrows). Cells are arranged in disorder with a slightly changed cell polarity. Cytoplasmic swelling, neuron loss, and microglia hyperplasia (white arrows) can also be seen (Figure 2(b)). In WNK- or EGB761-treated brains, neurons display smoothly stained regularly spaced pear-shaped cell bodies, containing large, round, or oval nuclei, similar to the nontransgenic ones, while cytoplasmic swelling and vacuolation are observed (arrow heads) (Figures 2(c) and 2(d)).

To investigate possible neuropathological correlates of decreased hippocampus-dependent spatial and associative learning and memory in APP/PS1 transgenic mice, histopathological analyses of brains were completed on a representative group of mice after behavioral studies, analyzing five serial sections across the CA1 area of hippocampus. Principal neurons appeared regularly spaced within the layers with stained cytoplasm and stained nuclei that were located approximately at the center of the soma. Cytoplasmic and nuclear membranes appeared to be smooth and uninterrupted, supporting round or oval forms of the nuclei, the characteristic pear-shaped form of pyramidal neurons (Figure 2(a)). In transgenic ones, cells were arranged in disorder with a slightly changed cell polarity. Although most of the neurons displayed the same morphological characteristics as those seen in the control brains, there was a small but distinct subpopulation of cells that showed signs of neurodegeneration, such as darkly stained and exhibited shrunken and triangulated neuronal body. Notably, cytoplasmic swelling, vacuolation, and rare microglia hyperplasia surrounding some neurons were also detected (Figure 2(b)). In WNK- and EGB761-treated mouse brains, cytoplasmic and nuclear membranes appeared to be smooth and uninterrupted, similar to those in nontransgenic brains, while few cytoplasmic swelling and vacuolation were detected (Figures 2(c) and 2(d)).

### 3.3. Evaluation on Brain A*β*-Deposits Burden

**Figure 3:**
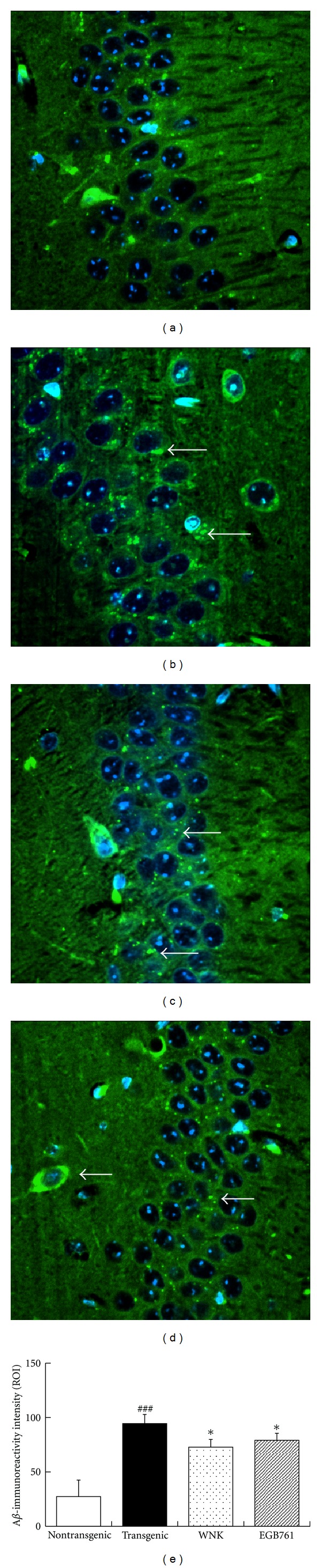
Immunofluorescent micrographs of amyloid plaques (×400). (a) Nontransgenic group; (b) transgenic group; (c) WNK group; (d) EGB761 group; (e) A*β*-immunoreactivity intensity of amyloid plaques. Significant A*β* plaques (arrows) can been seen in CA1 hippocampal neurons of an 8-month-old APP/PS1 mice (Figure 3(b)) while are occasionally found in those of wide-type littermate mice at the same age (Figure 3(a)). Overexpression of A*β* reduce after 3-month WNK or EGBA761 treatment (Figures 3(c), 3(d), and 3(d)). Note: ^###^ *P* < 0.001, compared with nontransgenic group; *P < 0.05, compared with transgenic group.

A*β* positive spots were significantly exhibited in CA1 hippocampal neurons of transgenic brains, while few were observed in nontransgenic brains (Figures 3(a) and 3(b)). Analysis data showed that the distribution of A*β* immunoreactivity in the hippocampal areas was significantly upregulated in transgenic mice when compared to age-matched wild-type controls (*P* < 0.001; Figure 3(e)). After 3 months, A*β* immunoreactivity was significantly reduced due to the consecutive WNK and EGB761 treatment as compared to APP/PS1 controls (*P* < 0.05, Figures 3(c), 3(d), and 3(e)).

### 3.4. Ultrastructure Observation

**Figure 4:**
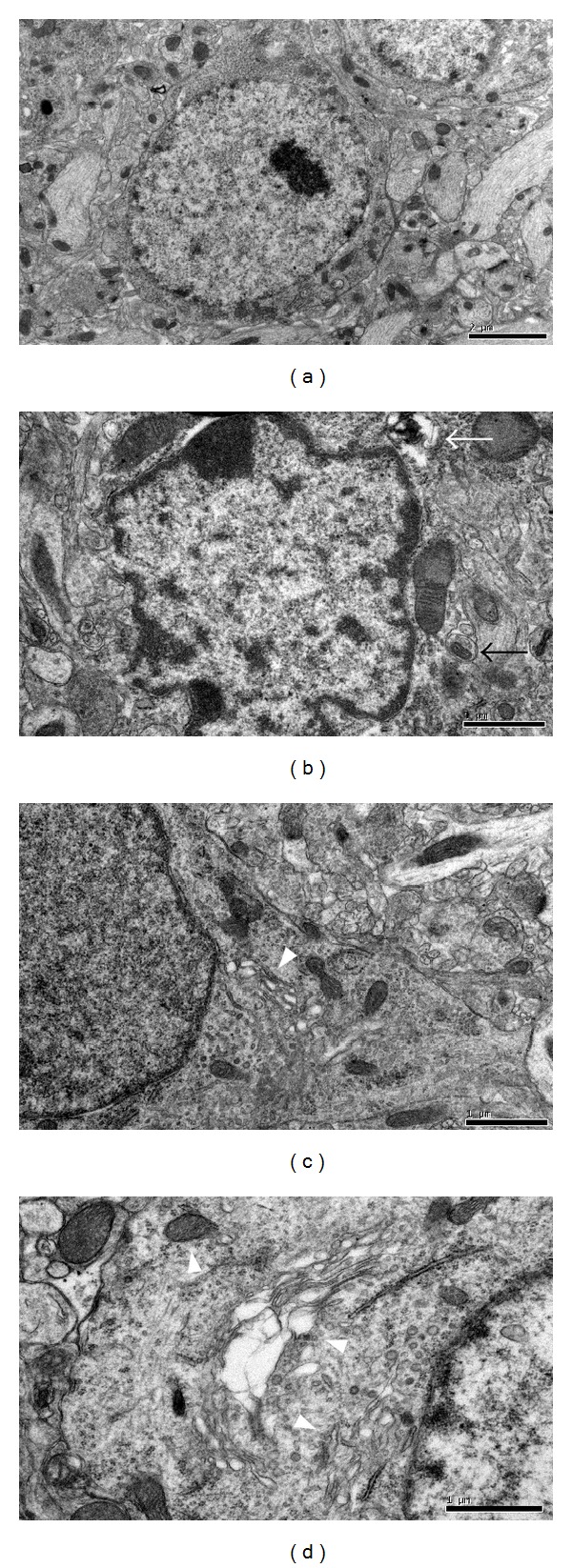
Electron micrographs of CA1 hippocampal neurons in mouse brains. (a) Nontransgenic group; (b) transgenic group; (c) WNK group; (d) EGB761 group. CA1 hippocampal neurons in nontransgenic mouse brain are well developed with normal organelles including nucleus (Figure 4(a)). In transgenic brain, extremely damaged structures display apoptotic death. Dilated rough endoplasmic reticulum, dilated mitochondria with fewer cristae inside, autophagosomes (black arrows), and vacuolation (white arrows) can also be seen in neurons (Figure 4(b)). After 3-month WNK or EGBA761 treatment, most neurons appeared to be with similar structure to nontransgenic ones, except for some deformed organelles, such as dilated rough endoplasmic reticulum, dilated Golgi complex, and destroyed mitochondria (arrow heads) (Figures 4(c) and 4(d)). Scale bar = 1~2 *μ*m.

This study was limited to the CA1 hippocampal region. In nontransgenic brains, no observe indications of neurodegeneration were observed. Most of the neurons with normal morphology (large, round, or oval nuclei, synaptic contacts on the soma, well-developed rough endoplasmic reticulum, and Golgi complex) had smooth cytoplasmic membranes marked only infrequently by small irregular infoldings. Rough endoplasmic reticulum was identified by the presence of ribosomes on its surface, mitochondria appeared as electron-dense oval structures with regular cristae inside and nuclei displayed smooth membranes (Figure 4(a)). Different from many cells with apparently normal morphology, in APP/PS1 transgenic mouse brains, some neurons and their organelles appeared to have undergone transformations, such as dilated rough endoplasmic reticulum and dilated mitochondria with fewer cristae inside, or even both cytoplasmic and nuclear membranes showing deep infoldings (Figure 4(b)). Few of the damaged structures displayed specific chromatin heterocondensation and marginalisation, typical of apoptotic death [36]. In WNK- and EGB761-treated brains, many neurons appeared to be with similar morphology to nontransgenic ones. Except for occasionally deformed mitochondrions recognized by remnants of cristae, rare autophagosomes, and dilated Golgi complex and reticulum, no additional organelles could be identified abnormal (Figures 4(c) and 4(d)).

## 4. Discussion

The early clinical features of AD are progressive learning and memory function deterioration. In this study, APP/PS1 mice (8 months of age) exhibited a significant impairment in spatial learning and a significant impairment of spatial memory retention in the MWM performance. This finding is consistent with the literatures on spatial learning and memory in this AD mouse model [34, 37]. Cognitive deficits in these mice correlate with onset and progression of AD-like pathology, indicating an association between the aggregation of A*β* peptide, and learning and memory impairments. Besides, APP/PS1 mice exhibited a significant increased hippocampal A*β* plaques loading in this study, while the plaques decreased after 3-month WNK or EGB761 treatment. In support of the immunohistochemical findings, histopathological and ultrastructural analysis of neurons in hippocampal CA1 area revealed more details. The degeneration changes were seen in 8-month APP/PS1 mouse brains, exhibiting darkly stained and shrunken and triangulated neuronal body, as well as gliosis surrounding some neurons in the brains. The degeneration was also characterized on the basis of ultrastructural appearance, especially showing as typical apoptotic death. Consistent with evidence for induced apoptosis in neurons, the reduced performance in hippocampus-dependent MWM with degenerative morphological and ultrastructural changes in CA1 area of hippocampus collectively supports hippocampus-dependent cognitive dysfunction in APP/PS1 mice. Simultaneously, the enhanced MWM performance of WNK and EGB761, as well as the eliminated A*β* plaques and improved neuron status, gives more evidence for their neuroprotective effects in this animal model. The possibility that WNK has a more obvious impact on cognitive deficits than EGB761, especially on the performance in the hidden platform task, might be due to the synergistic effect of ginseng, ginkgo and saffron extracts, since all three herbal extracts have been reported to have cognition-improving effect in different paradigms. Concordantly, findings in this study confirmed the protective effects of ginkgo, ginseng, and saffron against cognitive deficits *in vivo* [23, 38, 39].

 The amyloid hypothesis of AD posits that excess A*β* leads to dementia [40]. The abnormal accumulation of the Alzheimer's A*β* peptide is believed to play a pivotal, if not causal, role in AD. The development of learning impairment in APP/PS1 mice is supposed to correlate with age-dependent increases in A*β* levels in brain. Therefore, to better understand whether subjecting APP/PS1 transgenic mice would lead to changes in A*β* levels* in vivo*, we analyzed the hippocampal A*β* plaque distribution in APP/PS1 mice. Significant amounts of parenchymal plaques were detected in transgenic mice, consistent with a previous report that APP/PS1 transgenic mice had elevated levels of A*β* in several brain regions than normal littermates at 8 months of age [41].

Although data in this study demonstrated that A*β* plaque burden in brain is a candidate for mediating the beneficial effects of WNK or EGB761, there are conflict results arguing this influence on A*β*. A previous study concluded that ginkgo could not interfere with fibril formation *in vivo*, despite report that ginkgo decreased A*β* production* in vitro*, which is consistent with our result [42, 43]. Another study indicated that EGB 761 and its flavonoid fraction (CP 205) could prevent the A*β* fibril formation *in vitro*, too [44]. Furthermore, study on ginseng and its extracts, ginsenoside Rg1, Rg3, and Re, demonstrated significant reductions in the amount of A*β* detected in the brains of Tg 2576 after single oral doses of these agents [13]. Likewise, the inhibitive effect of saffron constituents on aggregation and deposition of A*β* had also been reported in the human brain [22].

For these contradictory findings, there might be some candidate reasons causing this bias. A possibility is that different animal models were adopted in these studies, which might lead to opposite results due to the animals' different pathological features. Alternative possibility is that since WNK is a combination of three herbal extracts, its effects might be a harmonious effect, antagonistic and synergistic, of all the active fractions in these extracts. Of course, to definitely demonstrate this hypothesis, more data needs to be presented. In this study, the finding from immunohistological determination of A*β* serves to confirm the possibility of an effect of WNK upon A*β* plaque deposition.

Furthermore, WNK's protective effect on morphology and ultrastructure in neurons of APP/PS1 transgenic mouse brain augments the temptation to invoke a neuroprotective mechanism to account for the results observed here. However, the present data can only be interpreted speculatively, due in part to the absence of some additional measures of interest. Further studies might be helpful to better demonstrate WNK's effect on A*β* toxicity in future, such as quantitation of cortical and hippocampal A*β*_1–42_ and A*β*_1–40_, neuronal pathology, total *β*-amyloid precursor protein, or some other splice variant of *β*-amyloid precursor protein.

## 5. Conclusions

AD represents the most common cause of dementia in the elderly and has a global prevalence of 6% in people over the age of 65 [45]. It is estimated that 1 in 85 persons worldwide will be affected by AD in 2050 [46]. Yet successful treatment for AD remains to be developed. Abundant clinical and preclinical evidence suggests that due to their long-term clinical application and complicated constituents, traditional Chinese medicines might beneficially affect various systems with less side effects and more clinical results compared with synthetic drugs.

The present study demonstrates the neuroprotective effects of WNK on enhanced spatial learning and memory in 8-month-old APP/PS1 transgenic mice, which might be dependent of an influence upon A*β* plaque burden. Findings in this study also indicated that the efficacy of WNK to affect cognitive function might be due to its effects on ameliorating the histopathological and ultrastructural consequences of CA1 hippocampal neurons in this animal model of AD. The data reported here support recent findings indicating the treatment with extracts of ginseng, ginkgo, and saffron extracts in AD patients [47–49]. Further efforts, such as elucidating the relationships between A*β* overexpression, inflammatory response, and neuronal autophagic stress, will greatly facilitate the interpretation of a multicenter international clinical trial of WNK currently under way and may enhance the design of future trials.

## Figures and Tables

**Table 1:** Concentrations of active components of WNK.

Active components	Concentrations (%)
Ginsenoside Rg1	5.5
Ginsenoside Re	3.2
Ginsenoside Rb1	13.1
Flavonoids	28.7
Total terpene lactones	11.6
Ginkgolide A	2.9

## References

[1] Price DL, Tanzi RE, Borchelt DR, Sisodia SS (1998). Alzheimer’s disease: genetic studies and transgenic models. *Annual Review of Genetics*.

[2] Holmes C, Wilkinson D (2000). Molecular biology of Alzheimer’s disease. *Advances in Psychiatric Treatment*.

[3] Savonenko A, Xu GM, Melnikova T (2005). Episodic-like memory deficits in the APPswe/PS1dE9 mouse model of Alzheimer’s disease: relationships to *β*-amyloid deposition and neurotransmitter abnormalities. *Neurobiology of Disease*.

[4] Garcia-Alloza M, Robbins EM, Zhang-Nunes SX (2006). Characterization of amyloid deposition in the APPswe/PS1dE9 mouse model of Alzheimer disease. *Neurobiology of Disease*.

[5] Shemer I, Holmgren C, Min R (2006). Non-fibrillar *β*-amyloid abates spike-timing-dependent synaptic potentiation at excitatory synapses in layer 2/3 of the neocortex by targeting postsynaptic AMPA receptors. *European Journal of Neuroscience*.

[6] Yan P, Bero AW, Cirrito JR (2009). Characterizing the appearance and growth of amyloid plaques in APP/PS1 mice. *The Journal of Neuroscience*.

[7] Klunk WE, Bacskai BJ, Mathis CA (2002). Imaging A*β* plaques in living transgenic mice with multiphoton microscopy and methoxy-X04, a systemically administered Congo red derivative. *Journal of Neuropathology and Experimental Neurology*.

[8] Amritraj A, Hawkes C, Phinney AL (2009). Altered levels and distribution of IGF-II/M6P receptor and lysosomal enzymes in mutant APP and APP + PS1 transgenic mouse brains. *Neurobiology of Aging*.

[9] Xu W, Zhan YQ, Huang W, Wang X, Zhang S, Lei H (2010). Reduction of hippocampal N-acetyl aspartate level in aged APPSwe/PS1dE9 transgenic mice is associated with degeneration of CA3 pyramidal neurons. *Journal of Neuroscience Research*.

[10] O’Hara M, Kiefer D, Farrell K, Kemper K (1998). A review of 12 commonly used medicinal herbs. *Archives of Family Medicine*.

[11] Gillis CN (1997). Panax ginseng pharmacology: a nitric oxide link?. *Biochemical Pharmacology*.

[12] Liu J, Cong W, Xu L, Wang JN (2004). Effect of combination of extracts of ginseng and ginkgo biloba on acetylcholine in amyloid beta-protein-treated rats determined by an improved HPLC. *Acta Pharmacologica Sinica*.

[13] Chen F, Eckman EA, Eckman CB (2006). Reductions in levels of the Alzheimer’s amyloid beta peptide after oral administration of ginsenosides.. *The FASEB Journal*.

[14] Allain H, Raoul P, Lieury A, LeCoz F, Gandon JM, D’Arbigny P (1993). Effect of two doses of Gingko biloba extract (EGb 761) on the dual-coding test in elderly subjects. *Clinical Therapeutics*.

[15] Rai GS, Shovlin C, Wesnes KA (1991). A double-blind, placebo controlled study of Ginkgo biloba extract (‘Tanakan’) in elderly outpatients with mild to moderate memory impairment. *Current Medical Research and Opinion*.

[16] Draebeck H, Petersen JR, Winberg N (1996). The effect of Ginkgo biloba in patients with intermittent claudicationm. *Ugeskrif for Laeger*.

[17] Kanowski S, Herrmann WM, Stephan K, Wierich W, Hörr R (1996). Proof of efficacy of the ginkgo biloba special extract EGb 761 in outpatients suffering from mild to moderate primary degenerative dementia of the Alzheimer type or multi-infarct dementia. *Pharmacopsychiatry*.

[18] Le Bars PL, Katz MM, Berman N, Itil TM, Freedman AM, Schatzberg AF (1997). A placebo-controlled, double-blind, randomized trial of an extract of Ginkgo biloba for dementia. *Journal of the American Medical Association*.

[19] Kleijnen J, Knipschild P (1992). Ginkgo biloba for cerebral insufficiency. *British Journal of Clinical Pharmacology*.

[20] Zhang Y, Shoyama Y, Sugiura M, Saito H (1994). Effects of *Crocus sativus* L. on the ethanol-induced impairment of passive avoidance performances in mice. *Biological and Pharmaceutical Bulletin*.

[21] Hosseinzadeh H, Sadeghnia HR, Ghaeni FA, Motamedshariaty VS, Mohajeri SA (2011). Effects of saffron (*Crocus sativus* L.) and its active constituent, crocin, on recognition and spatial memory after chronic cerebral hypoperfusion in rats. *Phytotherapy Research*.

[22] Papandreou MA, Kanakis CD, Polissiou MG (2006). Inhibitory activity on amyloid-*β* aggregation and antioxidant properties of *Crocus sativus* stigmas extract and its crocin constituents. *Journal of Agricultural and Food Chemistry*.

[23] Ghadrdoost B, Vafaei AA, Rashidy-Pour A (2011). Protective effects of saffron extract and its active constituent crocin against oxidative stress and spatial learning and memory deficits induced by chronic stress in rats. *European Journal of Pharmacology*.

[24] Abe K, Saito H (2000). Effects of saffron extract and its constituent crocin on learning behaviour and long-term potentiation. *Phytotherapy Research*.

[25] Ochiai T, Shimeno H, Mishima KI (2007). Protective effects of carotenoids from saffron on neuronal injury in vitro and in vivo. *Biochimica et Biophysica Acta*.

[26] Pitsikas N, Sakellaridis N (2006). *Crocus sativus* L. extracts antagonize memory impairments in different behavioural tasks in the rat. *Behavioural Brain Research*.

[27] Pitsikas N, Zisopoulou S, Tarantilis PA, Kanakis CD, Polissiou MG, Sakellaridis N (2007). Effects of the active constituents of *Crocus sativus* L., crocins on recognition and spatial rats’ memory. *Behavioural Brain Research*.

[28] Petkov VD, Kehayov R, Belcheva S (1993). Memory effects of standardized extracts of Panax ginseng (G115), Ginkgo biloba (GK501) and their combination Gincosan (PHL-00701). *Planta Medica*.

[29] Wesnes KA, Ward T, McGinty A, Petrini O (2000). The memory enhancing effects of a Ginkgo biloba/Panax ginseng combination in healthy middle-aged volunteers. *Psychopharmacology*.

[30] Wesnes KA, Faleni RA, Hefting NR (1997). The cognitive, subjective, and physical effects of a Ginkgo biloba/Panax ginseng combination in healthy volunteers with neurasthenic complaints. *Psychopharmacology Bulletin*.

[31] Mazza M, Capuano A, Bria P, Mazza S (2006). Ginkgo biloba and donepezil: A comparison in the treatment of Alzheimer’s dementia in a randomized placebo-controlled double-blind study. *European Journal of Neurology*.

[32] Snitz BE, O’Meara ES, Carlson MC (2009). Ginkgo biloba for preventing cognitive decline in older adults a randomized trial. *JAMA*.

[33] Lalonde R, Kim HD, Maxwell JA, Fukuchi K (2005). Exploratory activity and spatial learning in 12-month-old APP 695SWE/co + PS1/ΔE9 mice with amyloid plaques. *Neuroscience Letters*.

[34] Liu HL, Zhao G, Cai K, Zhao HH, Shi LD (2011). Treadmill exercise prevents decline in spatial learning and memory in APP/PS1 transgenic mice through improvement of hippocampal long-term potentiation. *Behavioural Brain Research*.

[35] Filali M, Lalonde R, Rivest S (2011). Subchronic memantine administration on spatial learning, exploratory activity, and nest-building in an APP/PS1 mouse model of Alzheimer’s disease. *Neuropharmacology*.

[36] Pliss L, Balcar VJ, Bubeníková V, Pokorný J, Fitzgibbon T, Št’astný F (2003). Morphology and ultrastructure of rat hippocampal formation after i.c.v. administration of *N*-acetyl-_L_-aspartyl-_L_-glutamate. *Neuroscience*.

[37] Trinchese F, Liu S, Battaglia F, Walter S, Mathews PM, Arancio O (2004). Progressive age-related development of Alzheimer-like pathology in APP/PS1 mice. *Annals of Neurology*.

[38] Lee ST, Chu K, Sim JY, Heo JH, Kim M (2008). Panax ginseng enhances cognitive performance in Alzheimer disease. *Alzheimer Disease and Associated Disorders*.

[39] Shif O, Gillette K, Damkaoutis CM, Carrano C, Robbins SJ, Hoffman JR (2006). Effects of Ginkgo biloba administered after spatial learning on water maze and radial arm maze performance in young adult rats. *Pharmacology Biochemistry and Behavior*.

[40] Hardy J, Selkoe DJ (2002). The amyloid hypothesis of Alzheimer’s disease: Progress and problems on the road to therapeutics. *Science*.

[41] Minkeviclene R, Banerjee P, Tanila H (2004). Memantine improves spatial learning in a transgenic mouse model of Alzheimer’s disease. *Journal of Pharmacology and Experimental Therapeutics*.

[42] Stackman RW, Eckenstein F, Frei B, Kulhanek D, Nowlin J, Quinn JF (2003). Prevention of age-related spatial memory deficits in a transgenic mouse model of Alzheimer’s disease by chronic Ginkgo biloba treatment. *Experimental Neurology*.

[43] Luo Y, Smith JV, Paramasivam V (2002). Inhibition of amyloid-*β* aggregation and caspase-3 activation by the Ginkgo biloba extract EGb761. *Proceedings of the National Academy of Sciences of the United States of America*.

[44] Longpré F, Garneau P, christen Y, Ramassamy C (2006). Protection by EGb 761 against *β*-amyloid-induced neurotoxicity: Involvement of NF-*κ*B, SIRT1, and MAPKs pathways and inhibition of amyloid fibril formation. *Free Radical Biology and Medicine*.

[45] Ferri CP, Prince M, Brayne C (2005). Global prevalence of dementia: a Delphi consensus study. *Lancet*.

[46] Brookmeyer R, Johnson E, Ziegler-Graham K, Arrighi HM (2007). Forecasting the global burden of Alzheimer’s disease. *Alzheimer’s and Dementia*.

[47] Akhondzadeh S, Sabet MS, Harirchian MH (2010). Saffron in the treatment of patients with mild to moderate Alzheimer’s disease: a 16-week, randomized and placebo-controlled trial. *Journal of Clinical Pharmacy and Therapeutics*.

[48] Ihl R, Tribanek M, Bachinskaya N (2010). Baseline neuropsychiatric symptoms are effect modifiers in Ginkgo biloba extract (EGb 761®) treatment of dementia with neuropsychiatric features. Retrospective data analyses of a randomized controlled trial. *Journal of the Neurological Sciences*.

[49] Zhong J, Tian JZ, Zhu AH, Yang CZ (2007). Clinical study on a randomized, double-blind control of Shenwu gelatin capsule in treatment of mild cognitive impairment. *Zhongguo Zhongyao Zazhi*.

